# Influence of Aerobic Training on The Mechanics of Ventricular Contraction After Acute Myocardial Infarction: A Pilot Study

**DOI:** 10.5935/abc.20180049

**Published:** 2018-04

**Authors:** Giovani Luiz De Santi, Henrique Turin Moreira, Eduardo Elias Vieira de Carvalho, Júlio César Crescêncio, André Schmidt, José Antônio Marin-Neto, Lourenço Gallo-Júnior

**Affiliations:** Divisão de Cardiologia - Departamento de Clínica Médica, Hospital das Clínicas da Faculdade de Medicina de Ribeirão Preto - Universidade de São Paulo, São Paulo, SP - Brazil

**Keywords:** Exercise, Rehabilitation, Myocardial Infarction, Myocardial Contraction, Stroke Volume, Magnetic Resonance Imaging

## Abstract

The study of myocardial contractility, based on the new anatomical concepts that govern cardiac mechanics, represents a promising strategy of analysis of myocardial adaptations related to physical training in the context of post-infarction.

We investigated the influence of aerobic training on physical capacity and on the evaluation parameters of left ventricular contraction mechanics in patients with myocardial infarction.

Thirty-one patients (55.1 ± 8.9 years) who had myocardial infarction in the anterior wall were prospectively investigated in three groups: interval training group (ITG) (n = 10), moderate training group (MTG) n = 10) and control group (CG) (n = 10). Before and after 12 weeks of clinical follow-up, patients underwent cardiopulmonary exercise testing and cardiac magnetic resonance imaging. The trained groups performed supervised aerobic training on treadmill, in two different intensities.

A statistically significant increase in peak oxygen uptake (VO_2_) was observed in the ITG (19.2 ± 5.1 at 21.9 ± 5.6 ml/kg/min, p < 0.01) and in the MTG 18.8 ± 3.7 to 21.6 ± 4.5 ml/kg/min, p < 0.01). The GC did not present a statistically significant change in peak VO_2_. A statistically significant increase in radial strain (STRAD) was observed in the CG: basal STRAD (57.4 ± 16.6 to 84.1 ± 30.9%, p < 0.05), medial STRAD (57.8 ± 27, 9 to 74.3 ± 36.1%, p < 0.05) and apical STRAD (38.2 ± 26.0 to 52.4 ± 29.8%, p < 0.01). The trained groups did not present a statistically significant change of the radial strain.

The present study points to a potential clinical application of the parameters of ventricular contraction mechanics analysis, especially radial strain, to discriminate post-infarction myocardial adaptations between patients submitted or not to aerobic training programs.

## Introduction

The helical conformation of the myocardial fibers, anchored in the pulmonary and aortic rings, determines a heart rotation movement around its longitudinal axis and confers a maximum mechanical efficiency to the cardiac muscle. The magnitude and characteristics of the present phenomenon are sensitive to left ventricle segmental and global contractile alterations.^1,2^

The parameters of myocardial deformation analysis and ventricular rotation represent a promising strategy for the study of cardiac contractility, allowing a reliable analysis of the left ventricular contraction dynamics, based on the new anatomical concepts that govern cardiac mechanics.^1,2^

Aerobic physical training (AFT) after myocardial infarction (MI) improves cardiac output, peak oxygen uptake (VO_2_), autonomic function and peripheral metabolism. Exercise programs, based on variables obtained through stress tests, are considered beneficial and safe for patients in the context of post-IM.^3^

However, scientific papers that investigated the effects of TFA on post-MI ventricular remodeling process, particularly through cavitary volumes measurement, as well as by estimating cardiac function by left ventricle ejection fraction, in resting conditions, showed heterogeneous and inconsistent results.^4-7^

Cardiac magnetic resonance allows an integrated analysis of myocardial function with the underlying pathology. Myocardial deformation curves, obtained by cardiac magnetic resonance imaging, represent tools capable of identifying initial or subclinical alterations, both in the segmental function and in the global function of the left ventricle.^8^

The use of these new methodologies incorporated into cardiac magnetic resonance can have a potential application in the identification of incipient contractile alterations in the post-infarction myocardium related to physical training. In this sense, we do not find in the scientific literature, articles that have tried to document, by myocardial deformation parameters analysis and ventricular rotation, the effects of AFT in patients in the context of post-MI.

It was investigated the influence of TFA, prescribed in two different intensities, on physical capacity and the analysis parameters of myocardial deformation and ventricular rotation in patients with a diagnosis of MI.

## Methods

### Patients

Thirty patients, 55.1 ± 8.9 years, with the diagnosis of MI, were prospectively investigated after signing a informed consent form; randomized into three groups: moderate training (MTG), interval training (ITG) and control (CG).

The inclusion criteria established were: anterior wall MI with exclusive involvement of anterior descending artery proximal third and asymptomatic ventricular dysfunction with left ventricular ejection fraction < 50%. Patients who progressed with heart failure, sustained ventricular tachycardia, chronic obstructive pulmonary disease, chronic renal failure and orthopedic or neurological limitations for physical exercise were excluded.

The study was conducted in accordance with the Helsinki declaration. The research ethics committee of our Institution (nº 11612/2008) approved the consent form and study protocol.

### Cardiopulmonary exercise test

An exercise test was performed with uptake of gases expired by the CPX/D analyzer MedGraphics (Saint Paul, USA). BreezeEX software was used for the acquisition, processing and storage of cardiorespiratory variables. A modified Balke protocol was applied on treadmill, with speed 1.5 mph in the first minute, 2.5mph in the 2nd minute and fixed in 3.0 mph from the third minute, followed by increasing increments of the slope of 2% every minute until the effort is interrupted, due to physical exhaustion. Continuous cardiac monitoring was performed by the 13-lead modified Mason-Likar shunt system, and blood pressure was measured manually every minute during the exercise and recovery period.

### Cardiac magnetic resonance

Tests were performed on the Magneton Vision, Siemens, 1.5T (Erhlangen, Germany), with 25 mT circular polarization gradient coils. The sequence used was the fast gradient echo with steady state acquisition (TRUE_FISP) with parameters adjusted to optimize the signal-to-noise ratio. Flip angle = 10º, cut thickness = 8mm; interval between cuts = 0 mm; 13 phases of the cardiac cycle in a single cut, each expiratory apnea, always synchronized to ECG, becoming a cardiac cycle film with optimal temporal and spatial resolution. The images were obtained along the vertical axis (4 chambers) and the short axis to cover the entire extension of the left ventricle.

### Evaluation of myocardial deformation and ventricular rotation

The evaluation of myocardial deformation and ventricular rotation was performed by the Multimodality Tissue Tracking computer program (MTT, version 6.6.0, Toshiba, Japan) through the analysis of cardiac magnetic resonance images generated with pulse sequences Steady State Free Precession (SSFP)).

### Prescription of physical training

Patients randomized to the training groups were subjected to three supervised weekly sessions of aerobic exercise on a treadmill, for a period of 12 weeks.

Training sessions were constituted by the following phases: warm-up, with 5 minutes duration; conditioning, with load adjustments (speed and incline) to maintain the heart rate (HR) within the training zone for 30 minutes; and the descent with a duration of 5 minutes.

The intensity of the TFA, defined by training HR interval, was established from a percentage of peak HR reached in the cardiopulmonary exercise test.

The HR of training for the randomized patients for the MTG was calculated in the following way: the minimum HR was established as representative of 60% of the peak HR, while the maximum HR of training was the representative of 70% of the Peak HR reached in the cardiopulmonary exercise test.

Patients randomized to ITG performed TFA applying a model called 4x4, consisting of 4 periods of 4 minutes duration with training HR between 85 to 95% of the peak HR reached in the cardiopulmonary exercise test, interspersed with periods of active recovery time of 3 minutes duration with training HR between 60 to 70% of the peak HR reached in the cardiopulmonary exercise test.

### Statistic analysis

Data are expressed as mean ± standard deviation. A value of p < 0.05 was considered statistically significant. The analysis of the distribution of the data was verified with the Kolmogorov-Smirnov test. The reason for the use of nonparametric tests was that the distributions of the analyzed variables did not present Gaussian distribution. The Kruskal-Wallis test with the Dunn post-test was used for intergroup comparison. The *Wilcoxon* rank-*sum test* was used for intragroup comparison. The statistical analysis was carried out with the SPSS 10.0 program (SPSS Inc., Chicago Illinois, USA).

## Results

The comparative analysis between the groups did not present statistically significant differences for variables initial evaluation of exercise cardiopulmonary test.

In contrast to the CG, the trained groups presented, after the 12-week period of TFA, increase with statistical significance of peak VO_2_, peak ventilation-minute (VM) and peak pulse oxygen (PO_2_) (p < 0.05) (Table 1).

**Table 1:** Exercise Cardiopulmonary Test Variables

	CG (n = 10)		ITG (n = 10)		MTG (n = 10)	
	Before	After	Before	After	Before	After
VO_2_ peak (ml/kg/min)	18,2 ± 4,4	17,1 ± 4,6	19,2 ± 5,1	21,9 ± 5,6*	18,8 ± 3,7	21,6 ± 4,5*
VM peak (L/min)	55,9 ± 17,5	48,4 ± 15,9^†^	61,4 ± 20,6	72,2 ± 21,9*	62,1 ± 14,5	68,6 ± 15,5^†^
Basal PO_2_ (ml/systole)	4,3 ± 1,1	4,1 ± 0,8	3,75 ± 0,7	4,3 ± 0,9	4,5 ± 1,5	4,1 ± 1,0
PO_2_ peak (ml/systole)	11,7 ± 3,1	11,3 ± 3,1	11,6 ± 3,0	12,8 ± 2,5^†^	11,1 ± 1,1	12,3 ± 1,7^†^
RER	1,08 ± 0,08	1,08 ± 0,08	1,12 ± 0,11	1,19 ± 0,10	1,15 ± 0,07	1,19 ± 0,08
HR rest (bpm)	64,1 ± 12,8	65,6 ± 6,6	63,1 ± 9,9	62,1 ± 6,0	63,6 ± 11,6	64,8 ± 8,2
HR peak (bpm)	122,9 ± 28,3	123,1 ± 28,2	131,8 ± 20,6	133,2 ± 21,7	131,6 ± 12,3	129,0 ± 18,3
SBP (mmHg)	158,5 ± 22,4	159,5 ± 15,5	149,5 ± 25,2	146,5 ± 16,8	153,0 ± 20,1	145,2 ± 17,9
DBP peak (mmHg)	8,2 ± 0,6	8,4 ± 0,7	8,1 ± 0,6	8,0 ± 0,5	8,3 ± 0,7	8,1 ± 0,6
DP (bpm.mmHg)	19628 ± 5523	19422 ± 3870	19989 ± 5770	19596 ± 4468	20229 ± 3864	19566 ± 3990

VO_2_: oxygen uptake; VM: ventilation-minute; PO_2_: oxygen pulse; RER: respiratory exchange rate; HR: heart rate; SBP: systolic blood pressure; DBP: diastolic blood pressure; DP: double product.

* p < 0.01: different, in the comparative analysis before and after, after the period of clinical follow-up.

† p < 0.05: different, before and after comparative analysis, after the clinical follow-up period.

The comparative analysis between the groups did not present statistically significant differences for variables initial evaluation of cardiac magnetic resonance, myocardial deformation and ventricular rotation.

In contrast to the trained groups, the CG presented, after the 12-week period of clinical follow-up, a statistically significant increase in radial strain (STRAD) (p < 0.05) (Table 2).

**Table 2:** Cardiac Magnetic Resonance Variable

	CG (n = 10)		ITG (n = 10)		MTG (n = 10)	
	Before	After	Before	After	Before	After
FDV (ml)	156,6 ± 39,3	148,2 ± 34,1	174,8 ± 55,8	178,8 ± 44,9	143,8 ± 52,9	141,0 ± 45,5
FSV (ml)	91,6 ± 37,0	83,9 ± 38,3	96,3 ± 52,3	96,3 ± 36,3	82,6 ± 38,9	76,2 ± 36,5
FE (%)	43,9 ± 11,5	45,7 ± 14,4	47,0 ± 10,8	47,2 ± 6,8	44,6 ± 9,5	47,6 ± 10,4
STLONG (%)	-9,0 ± 5,4	-9,1 ± 6,2	-9,2 ± 4,7	-8,6 ± 4,6	-10,1 ± 4,5	-10,5 ± 4,5
STCIRC_B (%)	-15,5 ± 4,3	-17,8 ± 3,3	-17,0 ± 3,3	-17,2 ± 3,0	-14,2 ± 4,6	-14,9 ± 3,4
STCIRC_M (%)	-13,5 ± 4,5	-14,4 ± 3,6	-13,5 ± 3,6	-14,5 ± 3,5	-12,0 ± 2,1	-12,5 ± 2,4
STCIRC_A (%)	-10,5 ± 4,6	-12,2 ± 6,9	-10,3 ± 5,6	-11,5 ± 4,5	-11,2 ± 4,4	-12,5 ± 8,4
STRAD_B (%)	57,4 ± 16,6	84,1 ± 30,9^†^	63,3 ± 19,5	58,6 ± 18,8	67,9 ± 24,5	60,4 ± 25,5
STRAD_M (%)	57,8 ± 27,9	74,3 ± 36,1^†^	59,1 ± 21,3	58,5 ± 25,8	57,5 ± 21,0	55,6 ± 19,8
STRAD_A (%)	38,2 ± 26,0	52,4 ± 29,8*	41,8 ± 25,0	41,4 ± 19,4	38,3 ± 25,8	38,9 ± 17,9
ROT_B (°)	-2,2 ± 1,4	-2,3 ± 0,9	-1,6 ± 1,3	-1,5 ± 1,1	-1,9 ± 0,9	-2,3 ± 1,2
ROT_A (°)	3,2 ± 1,7	4,0 ± 3,4	4,3 ± 2,4	4,0 ± 2,0	3,9 ± 1,7	3,5 ± 2,1
TWIST (°)	5,4 ± 2,1	6,3 ± 3,3	5,9 ± 2,8	5,5 ± 2,0	5,9 ± 1,5	5,9 ± 2,5

FDV: final diastolic volume; FSV: final systolic volume; EF: ejection fraction; STLONG: overall longitudinal strain; STCIRC_B: basal circumferential strain; STCIRC_M: medial circumferential strain; STCIRC_A: apical circumferential strain; STRAD_B: basal radial strain; STRAD_M: medial radial strain; STRAD_A: radial apical strain; ROT_B: basal rotation; ROT_A: apical rotation; Twist: angular difference between apical rotation and basal rotation.

* p < 0.01: different, in the comparative analysis before and after, after the period of clinical follow-up.

† p < 0.05: different, before and after comparative analysis, after the clinical follow-up period.

## Discussion

We conducted a pilot study in order to evaluate the influence of TFA, prescribed in two different intensities, on the physical capacity and mechanical contraction of the left ventricle in the context of post-MI.

The main finding of this study was the documentation of a different behavior from the STRAD in the CG in comparison with the trained groups. This result is important insofar as it suggests that the myocardial deformation parameters may be more sensitive, in comparison with the classical parameters of evaluation of ventricular remodeling, in the identification of post-infarction myocardial adaptations between patients submitted or not to TFA programs.

We postulate that in order to improve the mechanical efficiency of the cardiac muscle, there was an adaptation of the post-infarction myocardium in the GC that required an increase in systolic thickening as a probable mechanism for maintaining adequate systolic volume and cardiac output in the resting state.

On the other hand, TFA may have contributed to compensatory adaptive mechanisms sensitive to radial strain analysis not to be triggered in trained groups as part of post-infarction myocardial adaptations, necessary to meet the metabolic and tissue demands in resting state.

From the perspective of parameters analysis of myocardial deformation and ventricular rotation, the interval aerobic training did not show significant changes in left ventricle contraction mechanics in comparison with continuous moderate aerobic training.

Throughout last decades, since the publication of Jugdutt et al,^9^ several scientific works emerged that attempted to evaluate TFA influence on ventricular remodeling process in post-MI context. Giannuzzi et al.,^4^ showed an increase in cardiac function and maintenance of cavitary volumes. Kubo et al.,^5^ observed an increase in cavitary volumes and maintenance of cardiac function. Giallauria et al.,^6^ documented maintenance of both cavitary volumes and cardiac function.

In the present study, the cavitary volumes and the cardiac function estimated by left ventricle ejection fraction did not present statistically significant changes. Moreover, in this way, they were not able to identify different patterns of ventricular remodeling in the training groups compared to the CG.

From the point of view of functional capacity, we observed a comparable increase of 14% in ITG and MTG VO_2_ peak. We used the 4x4 aerobic interval training model recommended in several studies for promoting expressive increases in VO_2_ peak, compared to continuous moderate aerobic training.^10,11^ However, we did not show a statistically significant difference between the ITG and the MTG VO_2_ peak, after the period of physical training.

We highlight that the present study data are corroborated by SAINTEX-CAD Study findings that showed a similar increase in physical fitness, comparing interval aerobic training versus continuous moderate aerobic training, in a large casuistry of patients with coronary artery disease.^12^

### Study limitations

It is known that HR increases linearly with VO_2_ within defined limits in the bands of 50 to 90% maximum VO_2_. However, in the present study, we could not establish a relationship between training intensity and ventilatory thresholds.

Finally, the area of fibrosis was not analyzed. The extension of fibrosis in the infarction area infarction can be an important determinant of the results of AFT in myocardial deformation parameters and ventricular rotation.

## Conclusions

The findings of this study point to a potential clinical application of ventricular contraction mechanics parameters analysis, notably radial strain, in discriminating post-infarction myocardial adaptations between patients submitted or not to aerobic training programs.
